# Supplementary material for: Automated optimized parameters for T-distributed stochastic neighbor embedding improve visualization and analysis of large datasets
Source: Nat Commun. 2019 Nov 28;10:5415. doi: 10.1038/s41467-019-13055-y (PMC6882880; doi:10.1038/s41467-019-13055-y)
Supplement: Supplementary file 1 — Supplementary Information [file 41467_2019_13055_MOESM1_ESM.pdf]

Automated Optimized Parameters for T-Distributed Stochastic Neighbor Embedding Improve Visualization and Analysis of Large Datasets

BELKINA ET AL.

SUPPLEMENTARY FIGURES

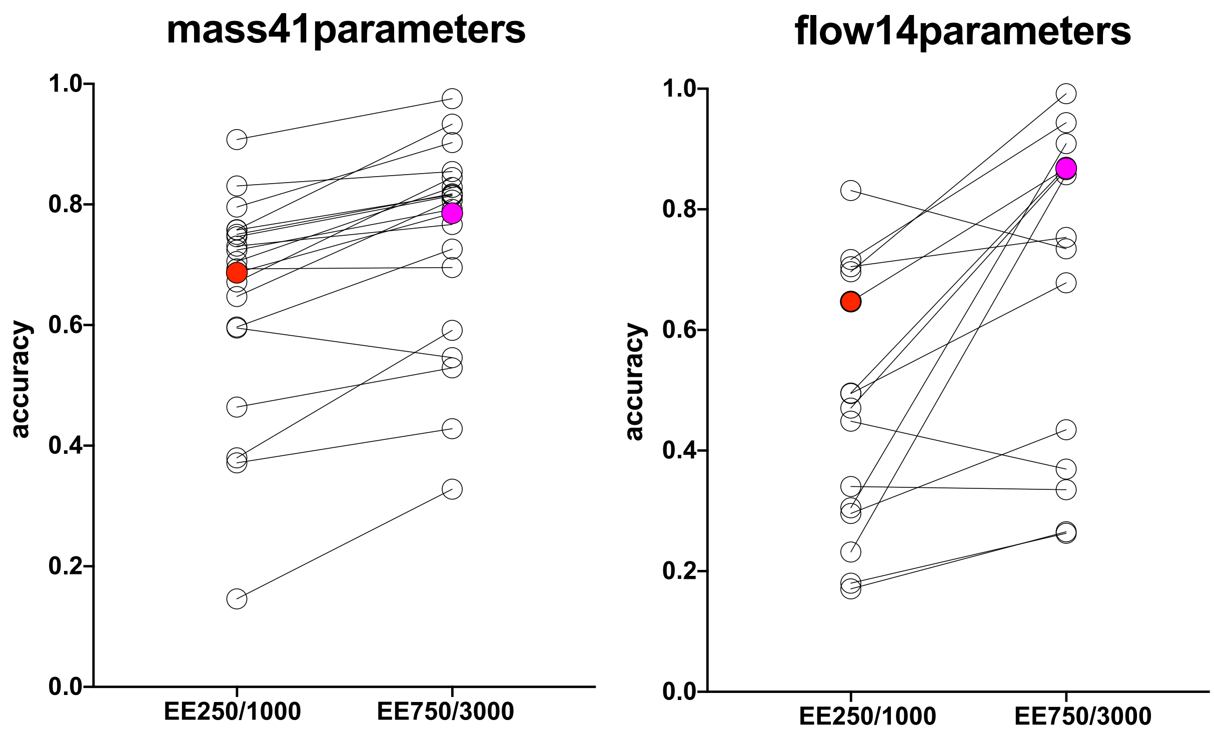

Supplementary Figure 1. 1NN accuracy of 'default' t-SNE embedding compared to 'extended' setup with longer EE. Accuracy scores for standard EE250/1000 and 'extended' EE750/3000 computation of mass cytometry (left) and flow cytometry (right) data per assigned class values (cell subsets as listed on Fig.1-2, open circles; overall scores, filled circles). Representative examples of multiple runs initiated with varying seed values are shown.

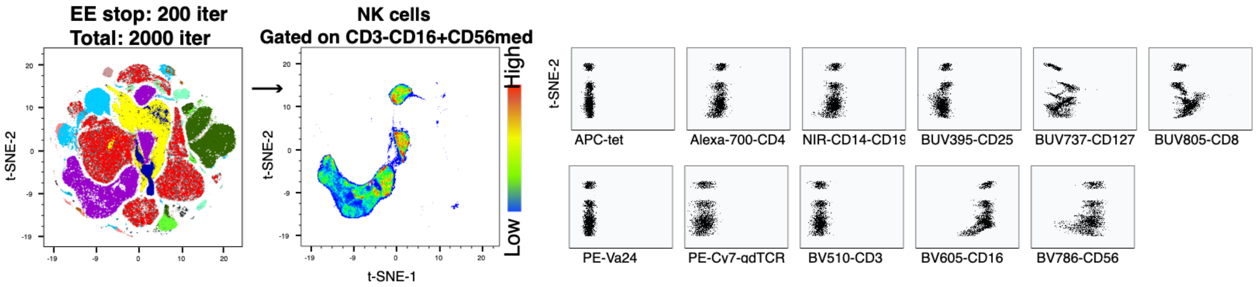

Supplementary Figure 2. Interrupted EE plateau results in cluster fragmentation. Clusters corresponding to CD3-CD16+CD56med NK cells were subsetted from the dataset and assessed as biaxial plots of different parameters plotted versus t-SNE-2 axis. Color overlays correspond to cell type classes labeled as in Fig. 2B (left) or density heatmap (middle).

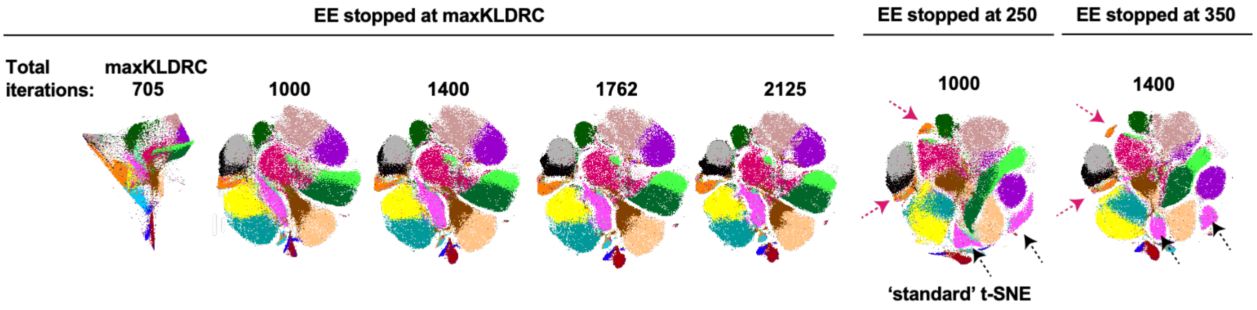

Supplementary Figure 3. KLD plateau effect on subset visualization in t-SNE maps. Mass cytometry data visualizations are shown as generated with varying duration of post-EE iteration time. Arrows mark cluster fragmentation. All color overlays correspond to cell type classes labeled as in Fig. 1-2. Representative examples of multiple runs with varying seed values are shown.

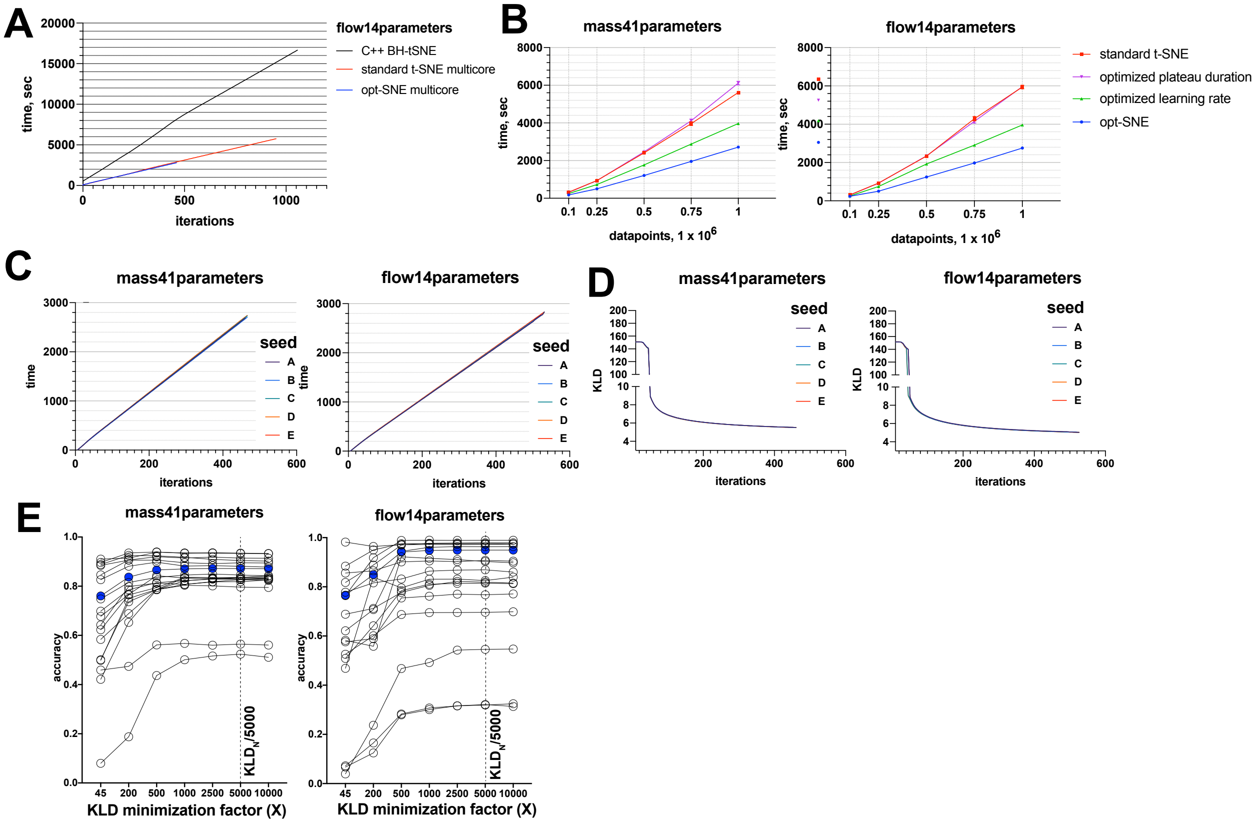

Supplementary Figure 4. Evaluation of opt-SNE embeddings. A, Speed benchmarks of original BH-tSNE (black), multicore BH-tSNE with standard settings (red), and multicore opt-SNE (blue). Representative examples of multiple runs with varying seed values are shown. B. Speed of t-SNE embedding (multicore BH-tSNE) with standard settings (red), optimized plateau duration/standard h = 200 (purple), optimized h/standard EE=250 iterations (green), and opt-SNE (blue). Average of 5 random seed runs for 5 dataset sizes (0.1-1 x 10^6 cells) of mass and flow cytometry data are plotted; error bars denote SEM. C, speed, and D, KLD graph of opt-SNE embeddings (multicore BH-tSNE) initiated from 5 random seed runs for 1 x 10^6 cells of mass (left) and flow (right) cytometry data are plotted; color indicates various seeds. E, 1NN accuracy scores for opt-SNE embeddings of of mass cytometry (left) and flow cytometry (right) data per assigned class values (cell subsets, open circles; overal scores, filled circles) for embeddings terminated based on various KLD minimization criteria X in ($KLD_{N-1} – KLD_N$) < KLD_N/X, with X plotted on horizontal axis. Representative examples of multiple runs initiated with varying seed values are shown.

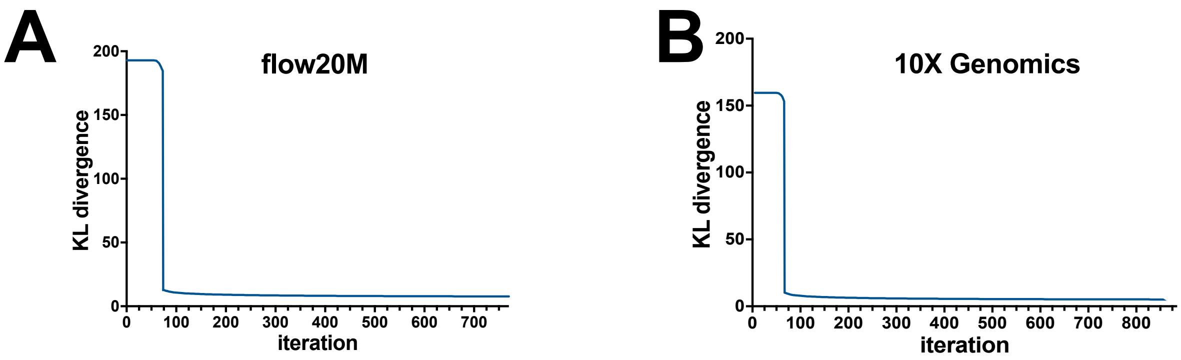

Supplementary Figure 5. KLD graphs for opt-SNE embedding of flow20M dataset (A) and 10X Genomics 1.3 million datapoints scRNA-seq dataset (B).

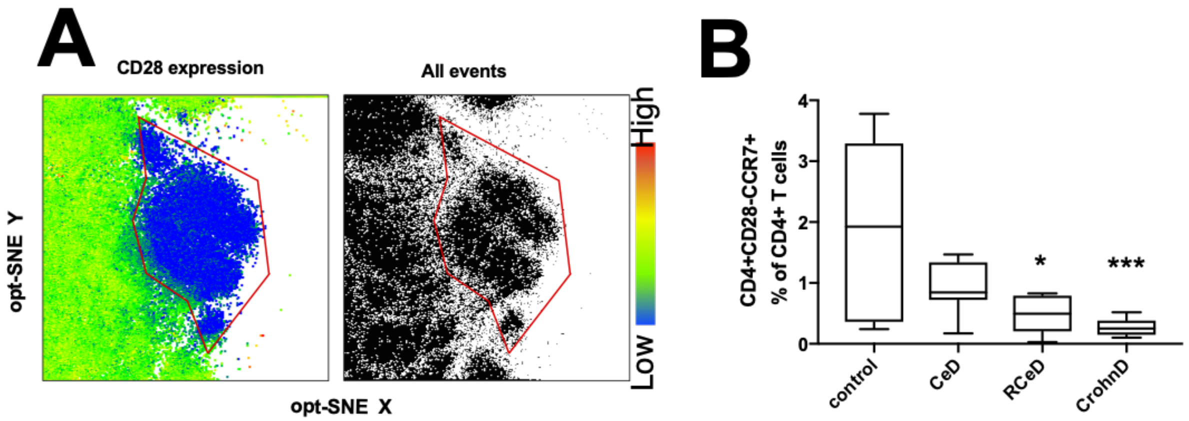

Supplementary Figure 6. A. Enlarged fragment of opt-SNE embedding showing CD4+CD28-CCR7+ cell cluster (left: color indicates CD28 expression intensity; right: dot plot). B. Frequencies of CD4+CD28-CCR7+ cells in CD4+ PBMC compared between different cohorts of subjects. CeD, Celiac disease; RCeD, refractory celiac disease; CrohnD, Crohn's disease. Two-tailed T tests were performed for statistical analysis. * $p = 0.0258$; *** $p = 0.0002$. All available datapoints are shown. Whiskers define min-max interval; central line defines median; box limits correspond to 25-75 percentiles.

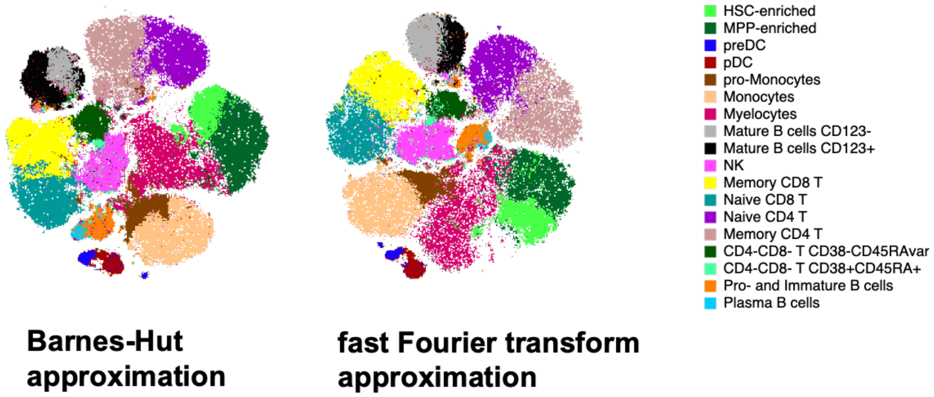

Supplementary Fig. 7. Opt-SNE embeddings of mass41parameter datasets with Barnes-Hut (left) and fast Fourier transform (right) used to compute approximation. Color overlays correspond to cell type classes.

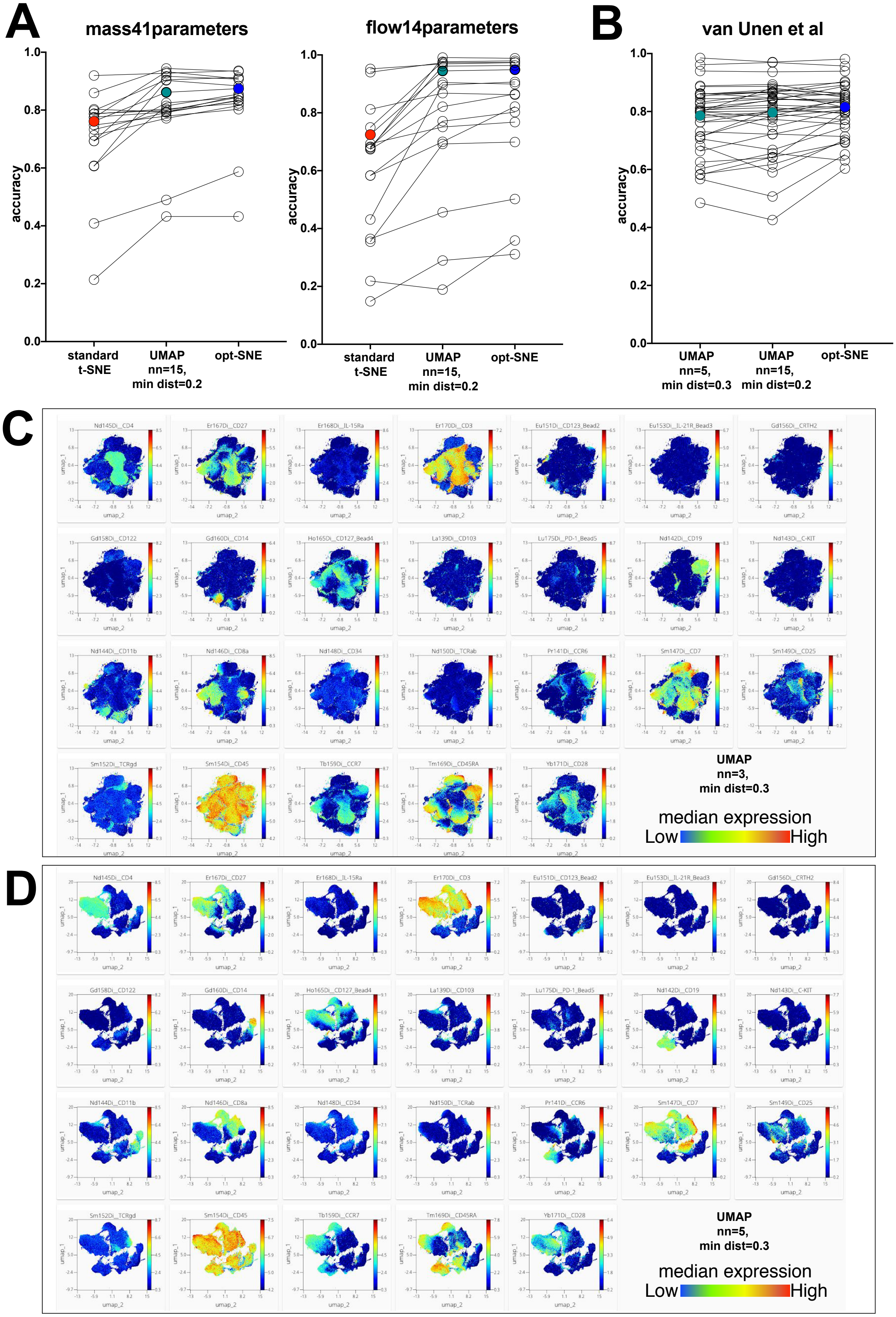
A
mass41parameters
flow14parameters
B
van Unen et al
accuracy
standard t-SNE
UMAP nn=15, min dist=0.2
opt-SNE
UMAP nn=5, min dist=0.3
C
UMAP nn=3, min dist=0.3
median expression
Low
High
D
UMAP nn=5, min dist=0.3
median expression
Low
High

E

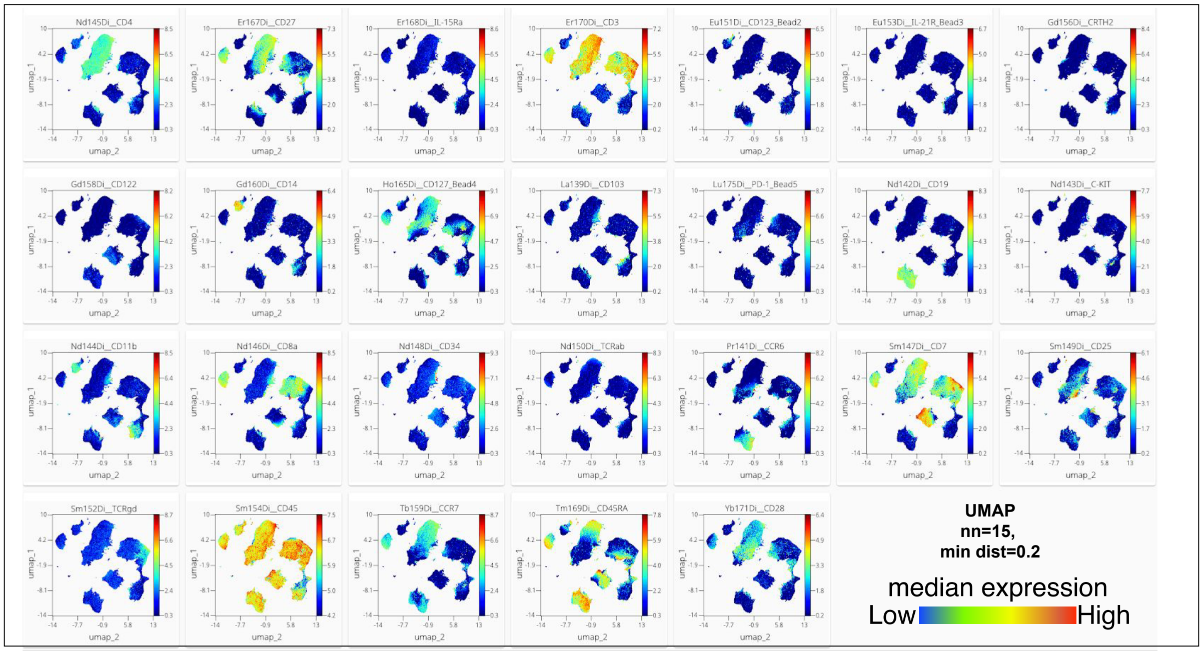

Supplementary Figure 8. NN accuracy comparison of standard t-SNE, UMAP and opt-SNE. A, Accuracy scores for gated cytometry data (biologically relevant subsets as listed in Fig.1-2, open circles; overall scores, filled circles). UMAP parameters were applied as indicated. A representative sample of 5 random seed runs is shown. B, van Unen et al[1] dataset was clustered with FlowSOM and accuracy scores for visualizations generated with indicated UMAP parameters as well as opt-SNE embedding are presented. Representative examples of multiple runs initiated with varying seed values are shown. C-E, UMAP embeddings visualized with mass cytometry labeling median intensities overlaid as color gradients.

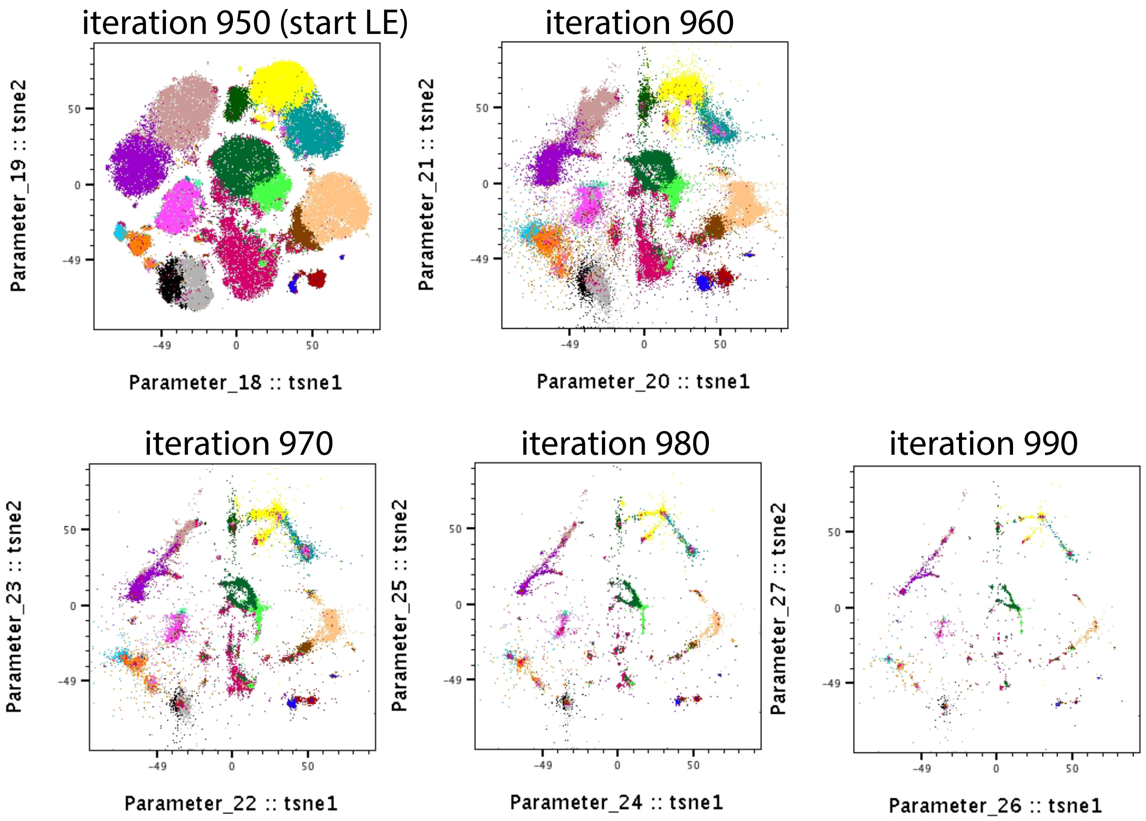

Supplementary Figure 9. Effect of late exaggeration (LE) on subset visualization in t-SNE maps. Mass cytometry data visualizations are shown as generated with varying duration of LE. N=200,000 events; initial learning step size = 16,000; EEF = 16; late exaggeraation factor = 16. All color overlays correspond to cell type classes labeled as in Fig. 1-2. Representative examples of multiple runs with varying seed values are shown.

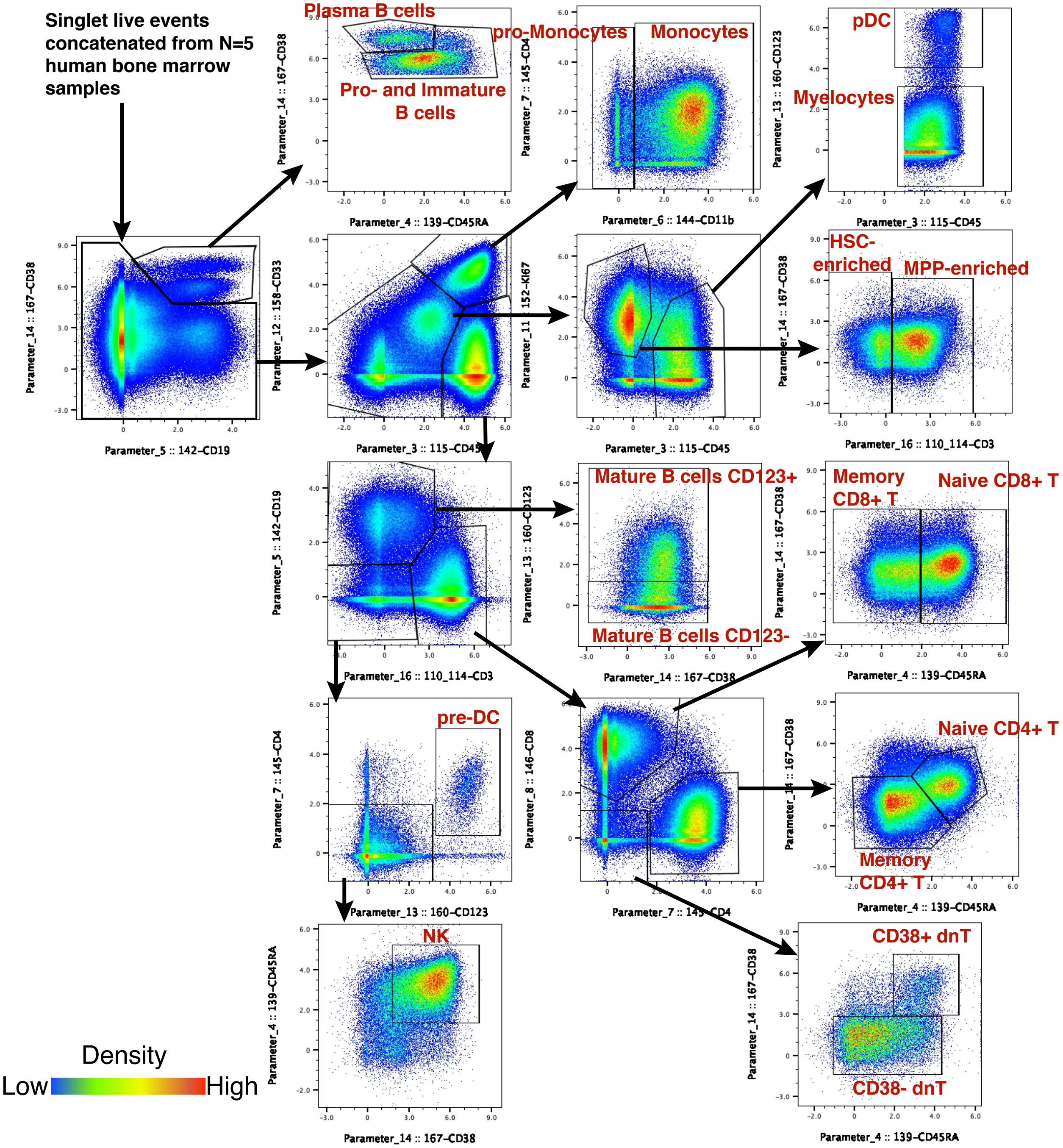

Supplementary Fig. 10. Mass cytometry manual data gating strategy based on Bendall et al[2].

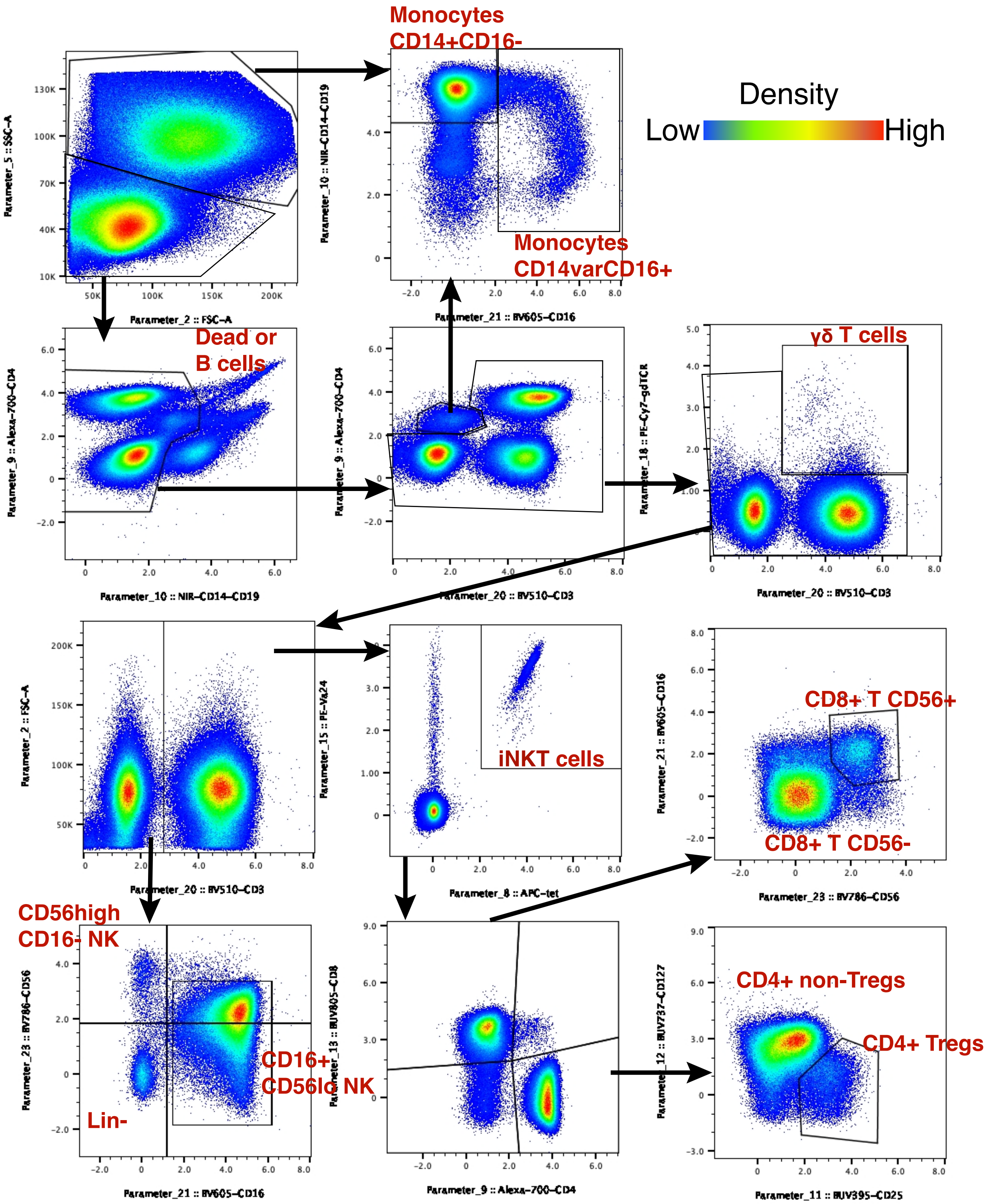

Supplementary Fig. 11. Flow cytometry manual data gating strategy based on Belkina et al[3].

Supplementary References

1. van Unen, V. et al. Visual analysis of mass cytometry data by hierarchical stochastic neighbour embedding reveals rare cell types. *Nat Commun* **8**, 1740 (2017).
2. Bendall, S.C. et al. Single-cell mass cytometry of differential immune and drug responses across a human hematopoietic continuum. *Science (New York, N.Y.)* **332**, 687-696 (2011).
3. Belkina, A.C. & Snyder-Cappione, J.E. OMIP-037: 16-color panel to measure inhibitory receptor signatures from multiple human immune cell subsets. *Cytometry A* **91**, 175-179 (2017).
